# Age-related changes in the response of retinal structure, function and blood flow to pressure modification in rats

**DOI:** 10.1038/s41598-018-21203-5

**Published:** 2018-02-13

**Authors:** Da Zhao, Christine T. O. Nguyen, Zheng He, Vickie H. Y. Wong, Anna K. van Koeverden, Algis J. Vingrys, Bang V. Bui

**Affiliations:** Department of Optometry and Vision Sciences, University of Melbourne, Parkville, 3010 Victoria Australia

## Abstract

Age-related changes to the balance between the pressure inside the eye (intraocular pressure, IOP) and the pressure inside the brain (intracranial pressure, ICP) can modify the risk of glaucoma. In this study, we consider whether the optic nerve in older rat eyes is more susceptible to acute IOP and ICP modification. We systematically manipulate both ICP and IOP and quantify their effects on ganglion cell function (electroretinography, ERG), optic nerve structure (optical coherence tomography, OCT) and retinal blood flow (Doppler OCT). We show that ganglion cell function in older eyes was more susceptible to a higher optic nerve pressure difference (ONPD = IOP – ICP). This age-related susceptibility could not be explained by poorer blood flow with elevated ONPD. Rather, as ONPD increased the retinal nerve fibre layer showed greater compression, and the retinal surface showed less deformation in older eyes. Our data suggest that age-related changes to connective tissues in and around the rat optic nerve make it less flexible, which may result in greater strain on ganglion cell axons. This may account for greater functional susceptibility to higher optic nerve pressure differences in older rat eyes. Further studies in a species with a well-developed lamina cribrosa are needed to determine the clinical importance of these observations.

## Introduction

Of the documented risk factors for glaucoma, ageing is perhaps the most robust. Aside from higher intraocular pressure (IOP), older age is the only other factor consistently and independently associated with increased risk of glaucoma development^1^ and progression of visual field loss^2^. Why age modifies the risk of ganglion cell injury is at present not entirely understood, as there appears to be little increase in IOP with advancing age^3^.

A number of mechanisms have been proposed to account for increased glaucoma risk with aging, including altered bioenergetics^4^, vascular impairment^5^, immune interactions^6^ and changes to optic nerve and peripapillary connective tissue biomechanical properties^7^. More recently, there has been renewed interest in the idea that other factors contributing to an increased pressure difference across the optic nerve and laminar cribrosa increase glaucoma risk. In particular, the tissue pressure immediately behind and surrounding the optic nerve; the fluid pressure from the subarachnoid space^8^, may be an important determinant of ganglion cell axon integrity. It has been proposed that lower intracranial pressure (ICP) with ageing leads to a higher pressure difference across the optic nerve head and thus increases the risk of glaucoma^9^. Consistent with this idea, those with primary open angle and normal tension glaucoma have been reported to have lower ICP (i.e. a higher translaminar pressure difference or translaminar pressure gradient) compared with age-matched controls and those with ocular hypertension^10,11^.

Morgan *et al*.^12^ observed in canine eyes that small changes in ICP can substantially change the shape of the optic nerve. In agreement with their data, we showed in rodents, although a species lacking a collagenous lamina cribrosa, that low and high ICP, increase and decrease IOP-induced tissue deformation and compression, respectively^13^. More importantly, we showed that changes in ICP affect the way that ganglion cells respond to IOP-related stress. In particular, the susceptibility of ganglion cell function, as measured using the electroretinogram (ERG), to IOP elevation was increased and decreased at low and high ICP, respectively^13^. More recently, Feola *et al*.^14^ using porcine eyes, report that modifying ICP affects the amount of strain exerted on optic nerve and retinal tissues, particularly the retinal nerve fiber layer (RNFL). Thus, an increased pressure difference across the optic nerve, regardless of whether it is induced by IOP elevation or ICP lowering, appears to be detrimental to ganglion cells.

Whilst the above studies suggest a role for ICP in modifying ganglion cell susceptibility to IOP elevation, such studies have employed younger eyes. Given that glaucoma is strongly associated with advancing age it is important to consider whether ageing alters interactions between ganglion cells, IOP and ICP. Using a range of approaches investigators have consistently found that the lamina cribrosa^15,16^ and sclera stiffens with age in human donor eyes^17–22^ as well as non-human primate^23^ and canine eyes^24^. A stiffer corneoscleral shell has been shown to result in larger IOP spikes^25–27^, leading to the idea that a more compliant shell can better absorb increases in IOP and should therefore protect the eye against IOP elevation. One would predict that in response to an elevated translaminar pressure difference (or optic nerve pressure difference (ONPD) in species with less well-developed lamina cribrosas), ganglion cells in older eyes should show increased susceptibility, which should be associated with increased tissue stiffness. In this study, we consider this hypothesis by comparing the effects of IOP elevation at low, normal and high ICP levels, on retinal function (electroretinography, ERG) and structure (optical coherence tomography, OCT) in 3 and 18 month old rats (equivalent to 10–15 and 45–50 human years^28^, respectively). Additionally, we related these functional and structural outcomes to a measure of inner retinal blood flow (Doppler OCT), in order to define vascular and mechanical contributions to age-related ganglion cell susceptibility to stress.

## Methods

### Animals

All experimental procedures were conducted in compliance with the National Health and Medical Research Council Australian Code of Practice for the care and use of animals for scientific purposes. Animal ethics approval was obtained from the Howard Florey Institute Animal Experimentation Ethics Committee (13-044-UM).

Experiments were undertaken on adult male Long-Evans rats aged 3 and 18 months (n = 5–9 each group). The lifespan of laboratory rats ranges from 2–3.5 years (average 3 years), thus 3 and 18 months approximates to 10–15 and 45–50 years of age for a human^28^, respectively. Animals were housed in a 21 °C environment in well-ventilated cages, with ad libitum access to normal rat chow and water. Lighting was regulated on a 12-hour light/dark cycle (50 lux, on at 8 am). All experiments were conducted under ketamine and xylazine anesthesia (60:5 mg/kg intraperitoneal injection, Troy Laboratory, Glendenning, NSW, Australia). Body temperature was maintained at 37.5 ± 0.5 °C using a circulating water heat-pad. Corneal anesthesia and mydriasis were achieved using drops of 0.5% proxymetacaine hydrochloride (Alcaine, Alcon Laboratories, Sydney, NSW, Australia) and 1% tropicamide (Mydriacyl, Alcon Laboratories), respectively.

### Intraocular Pressure modification

Intraocular pressure (IOP) elevation was achieved via a vitreous chamber cannula placed in one randomly chosen eye. As previous^29^, the cannula consisted of a 27 G needle (Fig. S1) connected to a saline reservoir (60 mL, Baxter International Inc. Toongabbie, NSW, Australia) via polyethylene tubing (0.8 mm outer, 0.4 mm inner diameter, Unomedical, Sydney, NSW, Australia). IOP was controlled by placing the reservoir to heights pre-calibrated against a manometer (Livingstone, Sydney, NSW, Australia).

### Blood Pressure monitoring

Blood pressure was monitored using a cannula placed in the femoral artery as previously described^29^. Briefly, a heparinized polyethylene cannula was inserted 3 cm proximally into the left femoral artery and secured to the surrounding tissue. The line was connected to a pressure transducer (Transpac, Abbott Critical Care Systems, Sligo, Ireland), whose signal (Bridge Amp ML 110, Amplifier ML 785, Powerlab/8SP, ADInstruments, Colorado Springs, CO, USA) gave direct and continuous BP monitoring (Lab Chart 7, ADInstruments).

### Intracranial Pressure modification

ICP was manipulated via a custom made dual-cannula placed into the lateral ventricle on the side ipsilateral to the cannulated eye^13^. The dual-lumen cannula consisted of a 23 G outer needle (0.6 mm diameter × 19 mm length, Becton Dickinson, Franklin, WI, USA) and a 30 G (0.3 mm diameter × 13 mm length, Becton Dickinson) inner needle, which were connect via polyethylene tubing (0.8 mm) to a pressure transducer (Transpac, Abbott Critical Care System) and a syringe pump (Pump 11 Elite Syringe Pumps, Harvard Apparatus, Holliston, MA, USA), respectively. This allowed for simultaneous ICP manipulation (Bridge Amp ML 110, Amplifier ML 785, Powerlab/8SP, ADInstruments) and recording (Lab Chart 7, ADInstruments). To prepare for lateral ventricle cannulation, rats were anesthetized and placed on a stereotaxic platform (Model 900, David Kopf Instruments, Los Angeles, CA, USA). A 2 cm by 2 cm flap of skin above the skull was removed. Connective tissue around the calvarial area was removed to expose the coronal sutures. Using a dental burr attached to a drill (Model 300, Dremel®, Robert Bosch Tool Corporation, Racine, WI, USA), a hole was drilled through the skull at 1.5 mm caudal to bregma and 2 mm lateral to the midline. The cannula was then inserted to a depth of 3.5 mm^30^.

### Experimental Protocol

Normal ICP for rats ranges from 4 to 7 mmHg^31–33^, thus we chose an ICP of 5 mmHg as our baseline. We also employ extremes of 25 mmHg and −5 mmHg as we had previously shown that such pressures produce differences in the response to IOP elevation when compared with the baseline ICP of 5 mmHg^13^.

Intracranial pressure was stabilized at −5, 5 or 25 mmHg for 20 minutes, prior to IOP elevation from 10 to 90 mmHg in 10 mmHg steps, with each step lasting 3 minutes. A 10 mmHg starting IOP was chosen as normal IOP in anesthetized rats ranges from 7–25 mmHg^34,35^. OCT or ERG assessment was conducted 2 minutes after the onset of each IOP step. Each animal underwent the IOP step protocol twice at two randomly chosen ICP levels (−5, 5 or 25 mmHg). Each IOP/ICP run was separated by 20 minutes, which was shown in pilot studies to provide sufficient time for functional and structural recovery from a preceding sequence of IOP elevation steps. OCT and ERG measurements were conducted in two parallel cohorts of animals (groups of n = 5–8, OCT; n = 5–9, ERG for each age and ICP level).

### Functional assessment: electroretinography

Retinal function was assessed using the full-field electroretinogram (ERG). As previous^36^, animals were dark-adapted overnight (12 hours). Care was taken to minimize light exposure during surgery, lateral ventricle cannulation and ERG setup to ensure maximum retinal sensitivity and to optimize ganglion cell specific scotopic threshold response (STR) measurements^37^. Responses were recorded using custom-made chlorided silver active and reference electrodes placed on the central cornea and sclera (ring shaped), respectively. A stainless steel ground electrode (F-E2-30, Grass Telefactor, West Warwick, RI, USA) was inserted subcutaneously into the tail.

Light was delivered using calibrated white LEDs (Luxeon LED, Philips® Lumileds Lighting Company, San Jose, CA, USA) embedded into a Ganzfeld sphere (Photometric Solutions International, Huntingdale, VIC, Australia). The stimulus and signal captured were triggered simultaneously with Scope™ software (ADInstruments). Signals (4 kHz sampling) were collected with filter settings of 0.3–1000 Hz (−3 dB) via pre-amplifiers (P511 Amplifier, Grass Telefactor) and saved (ML785 Powerlab 8SP, ADInstruments) for post-hoc analysis.

At each IOP ganglion/amacrine cell (scotopic threshold response or STR) function was assessed^37^, by taking the average of 20 flashes (2 second inter-stimulus interval) at a luminous exposure of −5.25 log cd∙s/m^2^. Waveforms were analyzed by measuring the amplitude from the peak of the positive STR component to the trough of the negative STR.

### Structural and blood flow assessment: Optical coherence tomography

Imaging was conducted under general ketamine-xylazine anesthesia and mydriasis. Throughout imaging, polyethylene glycol 0.4% with propylene glycol 0.3% eye-drops (Systane, Alcon Laboratories) were applied to moisten and improve the optical quality of the corneal surface. OCT scans were acquired using a spectral domain OCT (Envisu-R2200, Bioptigen, Leica Microsystems, Buffalo Grove, IL, USA) with a 50-mm rodent adaptor (1.7 mm field of view, working distance 5 mm). Two minutes following the onset of each IOP step, retinal volumes (1.4 × 1.4 × 1.57 mm) centered at the optic nerve head were acquired using 200 evenly distributed (in the vertical dimension) horizontal B-scans (1000 A-scans/B-scan). Following the volume scan a circle scan was employed to assess blood flow through the major vessels supplying the inner retina. The circle scan, consisted of 1000 A-scans, was positioned at 1 mm from the center of the optic nerve.

#### Structural Analysis

OCT images were extracted as TIFF stacks using the OCT Reader plugin (Bioptigen, Leica Microsystems) in FIJI (National Institutes of Health, Bethesda, MD, USA). Data represent the average of four B-scans closest to the center of the optic nerve. A masked operator manually segmented retinal layers using the Path Writer ImageJ PlugIn. RNFL thickness was measured from the anterior retinal surface (inner limiting membrane) to the inner margin of the inner plexiform layer. Total retinal thickness (TRT) was measured from the anterior (or inner) retinal surface to Bruch’s membrane. RNFL and TRT were averaged across retinal eccentricities 200 to 400 μm from the center of the optic nerve, from both the nasal and temporal retina. Deformation of the anterior retinal surface was determined by taking the difference between the average surface position and a peripheral reference plane demarcated by Bruch’s membrane at 2 mm nasal and temporal to the centre of the optic nerve. Changes in RNFL and TRT are expressed as relative to baseline (%) measured at 10 mmHg for that ICP level. Anterior surface deformation is given as a difference (in μm) from baseline measured at 10 mmHg.

#### Blood flow Analysis

Doppler blood flow images were exported as TIFFs for further analysis using ImageJ. A region of interest from the inner limiting membrane down to the outer border of the inner plexiform layer was selected. Background noise was reduced using the de-speckle function. The despeckle function replaces an individual pixel value with the median of itself and its adjacent neighbors (3 × 3 pixel median filter), which preserves boundaries better than a simple average filter. Color deconvolution was then used to separate the image into red, green and blue channels. The number of red and blue pixels was summed and expressed as a proportion of the region of interest. Finally, changes in relative blood flow are expressed relative to baseline (%).

### Statistical analysis

Statistical analysis was undertaken using GraphPad Prism (v. 6.01, La Jolla, USA). All experimental data underwent normality (Kolmogorov-Smirnov test) and homogeneity of variance assessment (Bartlett’s test & Levene’s test). Comparisons across age and IOP level were undertaken using two-way repeated measures ANOVA. Post-hoc analysis was conducted using Dunnett’s multiple comparison (within groups, IOP effect) and Tukey’s multiple comparison (across groups, ICP and ICP/IOP effect). Group data are given as mean ± SEM.

## Results

### Older eyes show greater dysfunction with pressure change

**Figure 1:**
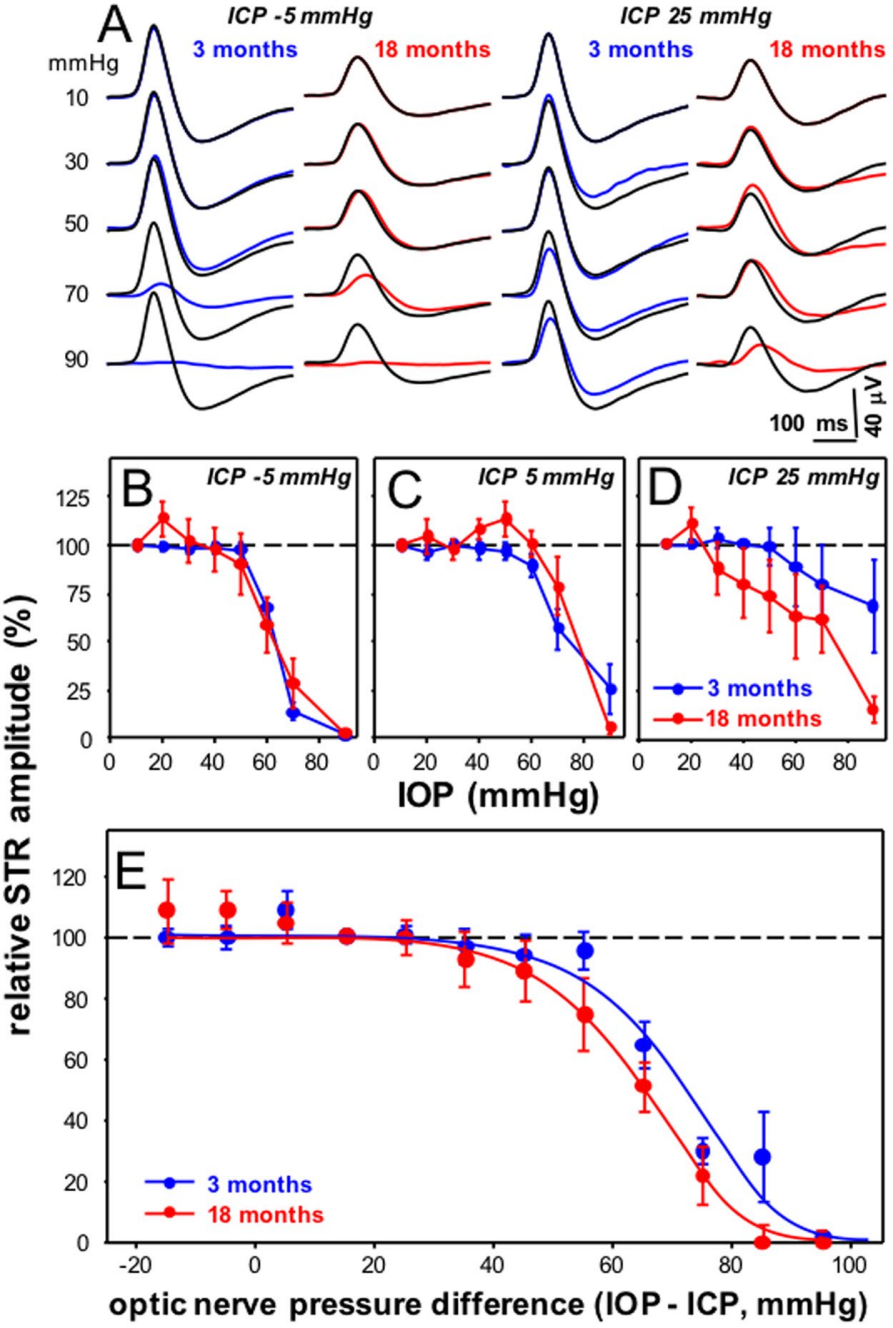
Effect of IOP and ICP modification on ganglion cell function in 3 and 18 month old rats. (**A**) Group average STR waveforms for young (3 month, blue traces) and older rat eyes (18 months, red traces) in response to increasing IOP at ICP of −5 and 25 mmHg. (**B–D)**. Group average (±SEM) normalized STR amplitude for young and older eyes as a function of IOP at ICP −5 mmHg (**B**, 3 month n = 6, 18 month n = 7) ICP 5 mmHg (**C**, 3 month n = 9, 18 month n = 7) and ICP 25 mmHg (**D**, 3 month n = 5, 18 month n = 6). (**E**) All data from panels B-D combined and plotted as a function of the optic nerve pressure difference (ONPD = IOP – ICP, mmHg). Lines indicate dose response curve fits to 3 and 18 month old data.

We first considered the effect of IOP and ICP modification on retinal ganglion cell function using the STR, which was elicited with very dim light levels. Figure 1A shows averaged waveforms for control and treated eyes from 3 and 18 month old rats at extreme ICP levels of −5 and 25 mmHg. Generally, STR amplitudes from 18 month old rats were smaller than were those from younger animals (18 vs 3 month baseline at ICP −5 mmHg: 18 ± 3 vs. 29.1 ± 3 µV; ICP 5 mmHg: 18 ± 4 vs. 21 ± 3 µV; ICP 25 mmHg: 16 ± 5 vs. 26 ± 2 µV, all P < 0.05). Higher IOP levels lead to attenuation of the STR in both 3 month (at ICP 25 mmHg; IOP 60 mmHg, 21.4 ± 5.0 µV; 70 mmHg, 19.3 ± 5.3 µV; 90 mmHg 18.4 ± 5.0 µV) and 18 month old rats (at ICP 25 mmHg; IOP 60 mmHg, 11.3 ± 4.5 µV; 70 mmHg, 9.9 ± 4.6 µV; 90 mmHg 2.8 ± 1.0 µV). This effect was more apparent at low ICP in comparison to the higher ICP level in both young (at ICP −5 mmHg; IOP 60 mmHg, 19.4 ± 2.1 µV; 70 mmHg, 8.7 ± 2.9 µV; 90 mmHg 0.5 ± 0.2 µV) and older eyes (at ICP −5 mmHg; IOP 60 mmHg, 7.7 ± 3.4 µV; 70 mmHg, 3.8 ± 2.6 µV; 90 mmHg 0.3 ± 0.4 µV). In 3 month old animals, IOP elevation to 90 mmHg reduced the STR by −98% ± 1% when ICP was −5 mmHg (Fig. 1B), whereas it was reduced by −32% ± 24% when ICP was 25 mmHg (P < 0.05, Fig. 1D). There was also greater IOP-induced attenuation of the STR response in 18 month old eyes compared with younger eyes (Fig. 1B–D). In particular, at the higher ICP level of 25 mmHg, IOP elevation to 90 mmHg reduced the STR by −85% ± 7% in 18 month old eyes, whereas this reduction was only −32% ± 24% in the 3 month old group (P < 0.05).

STR amplitude for each eye was normalized against its own baseline response, which was measured with IOP set at 10 mmHg. Group averaged data plotted (Fig. 1B–D) as a function of IOP confirmed that at the highest ICP, ganglion cell function in older eyes was more susceptible to IOP elevation than younger eyes (Fig. 1D). To better understand the relationship between IOP and ICP, data for all three ICP levels were normalized to a habitual optic nerve pressure difference (ONPD = IOP – ICP) of 15 mmHg (e.g. IOP 20 mmHg - ICP 5 mmHg). These data are plotted as a function of ONPD in Fig. 1E. Nonlinear fit with a sigmoidal function shows that older eyes had an EC50 (64.1, [95% CL, 60.6 to 67.7] mmHg) that was 5 mmHg more sensitive to the ONPD than younger eyes (71.3 [95% CL, 68.2 to 74.6] mmHg). The slopes of these functions were similar (3 month −0.054 [−0.070, −0.035] vs. 18 months −0.054 [−0.076, −0.032]).

### Older eyes show less blood flow attenuation

**Figure 2:**
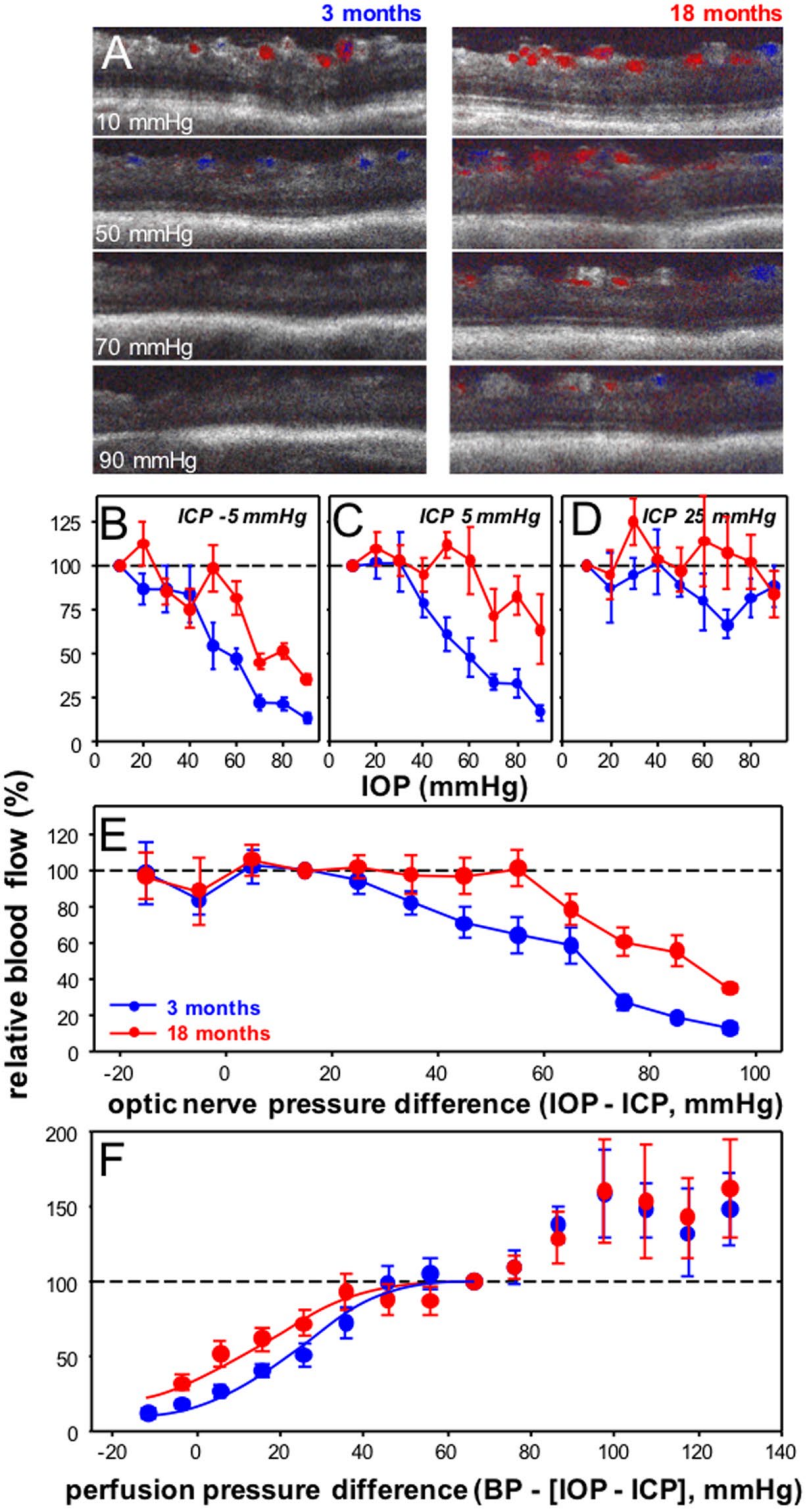
Effect of IOP and ICP modification on inner retinal blood flow in 3 and 18 month old rats. (**A**) Representative Doppler blood flow for a young (3 month, left panels) and an older eye (18 months, right panels) to increasing IOP levels at an ICP of −5 mmHg. Group average (±SEM) normalized blood flow (against baseline IOP = 10 mmHg) for young and older eyes as a function of IOP at ICP −5 mmHg (**B**, 3 month n = 5, 18 month n = 5), ICP 5 mmHg (**C**, 3 month n = 5, 18 month n = 5) and ICP 25 mmHg (**D**, 3 month n = 5, 18 month n = 5). (**E**) Data for each eye is normalized against an ONPD of 15 mmHg. All data are combined for young and older eyes and plotted as a function of ONPD (mmHg). (**F**) Data for each eye is normalized against a perfusion pressure difference of 70 mmHg. All data are combined for young and older eyes and plotted as a function of optic nerve perfusion pressure difference (BP − [IOP − ICP], mmHg). Lines indicate dose response curve fits to 3 and 18 month old data.

Blood flow images are shown in Figure 2. Comparison of a 3 and 18 month old eye suggest that blood flow through the major inner retinal vessels in the older eye was more resistant to IOP elevation compared with its younger counterpart. This appears to be particularly the case at low (Fig. 2B) and normal ICP (Fig. 2C). Two-way ANOVA comparisons between the two age groups ages across ONPD shows that blood flow in 18 month old eyes was less susceptible to higher ONPD than younger eyes (age effect F_1,218_ = 17.9, P < 0.001, ONPD effect F_10,218_ = 13.0, P < 0.001, interaction F_10,218_ = 0.93, P = 0.49).

At normal (3 month 82 ± 3 vs. 18 month 87 ± 4 mmHg, p = 0.33) and low ICP (3 month 84 ± 3 vs. 18 month 90 ± 4 mmHg, p = 0.16) levels there was no significant difference in blood pressure between young and old animals. However, with elevated ICP, blood pressure was significantly higher in older animals (131 ± 5 mmHg) compared with their younger counterparts (113 ± 5 mmHg, P = 0.02). Given this difference in the blood pressure response, blood flow in young and older eyes was also compared after taking into account changes in blood pressure induced by ICP manipulation, as shown in Fig. 2F. Two-way ANOVA comparisons between ages across the entire spectrum of optic nerve perfusion pressure differences indicated that while blood flow changed with perfusion pressure difference (F_12,199_ = 18.05, P < 0.001) there was no difference in blood flow between 3 and 18 month old eye as a function of perfusion pressure (age effect F_1,199_ = 1.96, P = 0.16, interaction F_10,218_ = 0.65, P = 0.80). However, if we consider only those perfusion pressures below baseline (i.e. < 70 mmHg) then there was a significant interaction between age and perfusion pressure (interaction F_7,168_ = 2.3, P = 0.03, age effect F_1,168_ = 4.9, P = 0.03). Sigmoidal curve fits confirm that blood flow was better in older eyes (EC50 (13.7 [95% CL, 7.2 to 20.4] mmHg) compared with younger eyes (27.7 [95% CL, 23.2 to 32.1] mmHg).

### Older eyes show more RNFL compression

**Figure 3:**
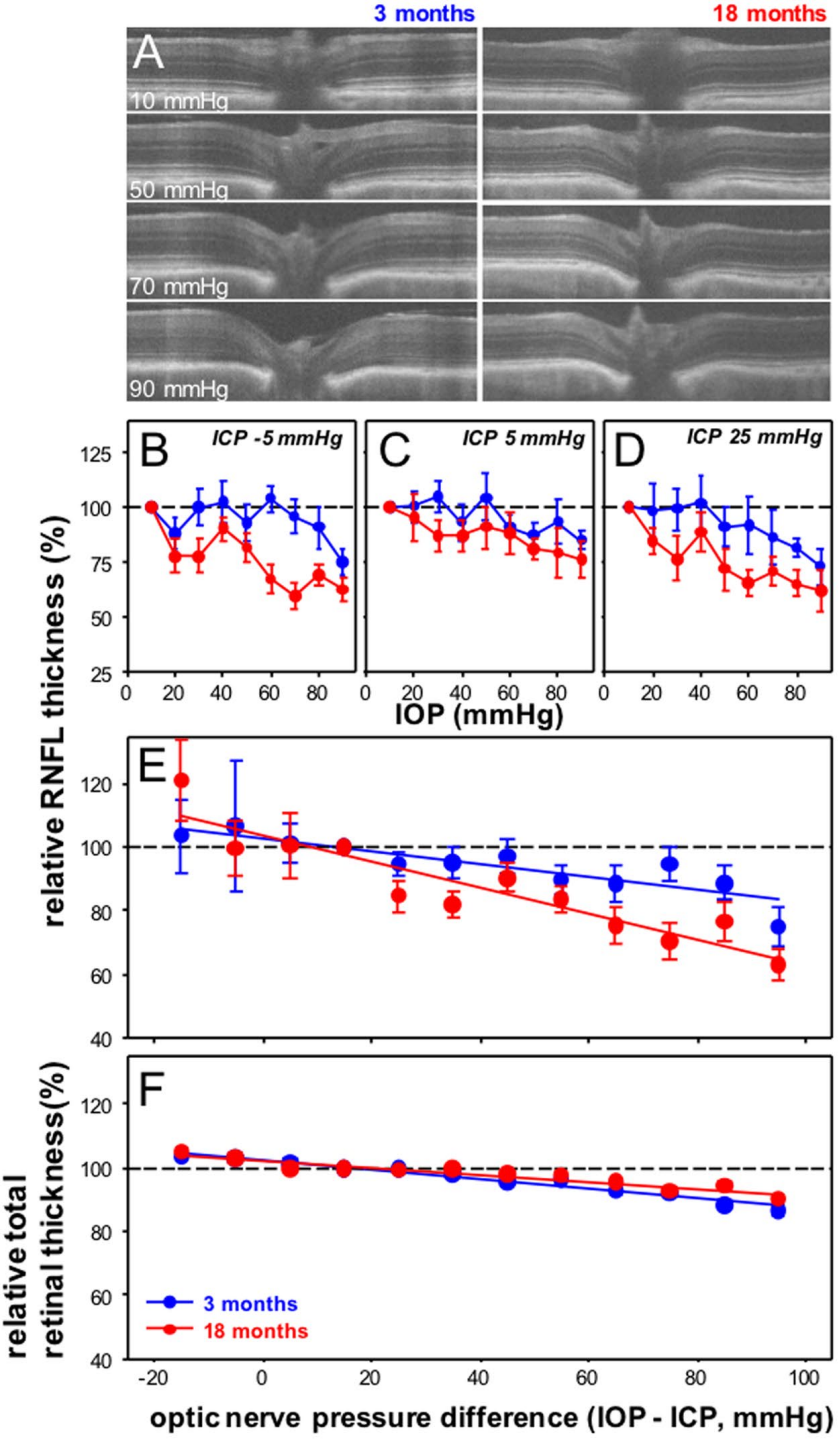
Effect of IOP and ICP modification on RNFL thickness in 3 and 18 month old rats. (**A**) Representative OCT cross-sections for a young (3 month, left panels) and an older eye (18 months, right panels) with increasing IOP at an ICP of −5 mmHg. (**B**–**D**) Group average (±SEM) normalized RNFL thickness for young and older eyes as a function of IOP at ICP −5 mmHg (**B**), 3 month n = 5, 18 month n = 8), ICP 5 mmHg (**C**, 3 month n = 5, 18 month n = 6) and ICP 25 mmHg (**D**, 3 month n = 5, 18 month n = 8). (**E**) All data from panels B–D combined and plotted as a function of ONPD (mmHg). Linear fits for 3 (y = −0.20*x + 103, r^2^ = 0.08, P < 0.001) and 18 month age groups (y = −0.44*x + 10, r^2^ = 0.19, P < 0.0017). (**F**). Relative total retinal thickness as a function of ONPD. Linear fits for 3 (y = −0.15*x + 103, r^2^ = 0.62, P < 0.001) and 18 month age groups (y = −0.11*x + 102, r^2^ = 0.39, P < 0.001).

Next we compared the effects of pressure modification on axial deformation of the anterior retinal surface and changes to the thickness of the RNFL and TRT between young and older eyes. Figure 3A shows representative OCT B-scan cross-sections through the center of the optic nerve for a 3 and 18 month old eye over a range of IOP levels. It is apparent that with higher IOPs, for regions near the optic nerve, the retina was thinner and the anterior surface of the retina was posteriorly deformed.

Quantification of change in RNFL thickness is shown in Fig. 3B–D. IOP-induced compression of the RNFL near the optic nerve was greater in older eyes at all ICP levels. RNFL compression for animals at all ICP levels was combined and plotted as a function of ONPD as shown in Fig. 3E. An increase in the ONPD resulted in significantly greater RNFL compression in older eyes (age effect F_1,271_ = 7.1, P = 0.008, ONPD effect F_10,271_ = 4.15, P < 0.001, interaction F_10,271_ = 0.82, P = 0.61).

Figure 3E shows that there was a linear relationship between RNFL compression and ONPD. The slope for older eyes was significantly steeper than younger eyes (3 month −0.19 [95% CI, −0.30, −0.08] vs. 18 month −0.39 [−0.50, −0.28], F = 5.8, P = 0.02). Compression of total retinal thickness in the same retinal location was conversely greater in the younger eyes (slopes 3 month −0.15%/mmHg [95% CI, −0.18, −0.13] vs. 18 month −0.11%/mmHg [−0.13, −0.09], F = 9.9, P < 0.01; Fig. 3F).

### Older eyes show less surface deformation

**Figure 4:**
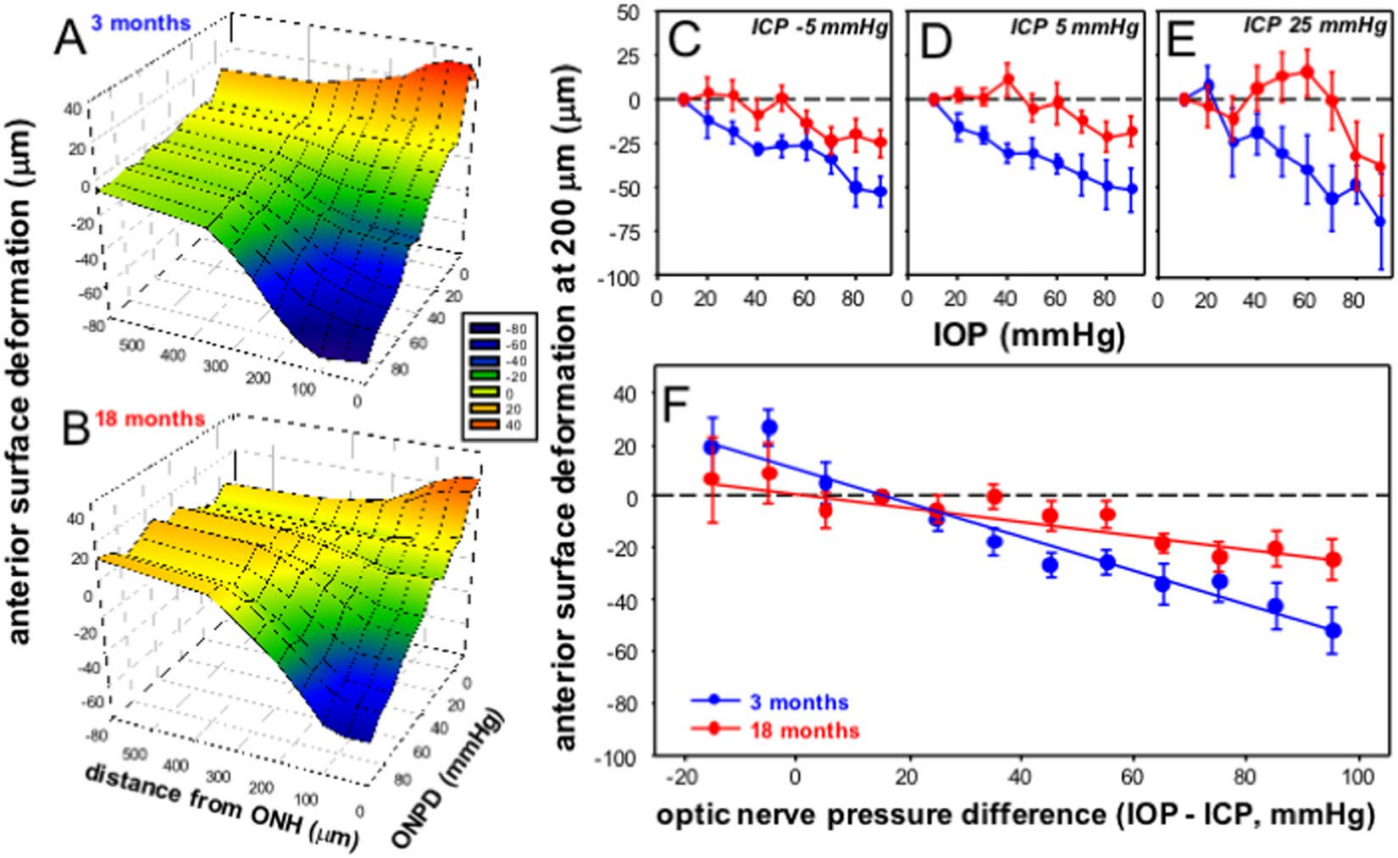
Effect of IOP and ICP modification on anterior retinal surface deformation (200 μm from the center of the optic nerve, relative to peripheral Bruch’s membrane plane) in 3 and 18 month old rats. Group average relative anterior surface deformation (axial change from baseline at 10 mmHg) plotted as a function of distance from the center of the optic nerve and ONPD for 3 (**A**) and 18 month old (**B**) rats. (**C–E**). Group average (±SEM) relative surface deformation (change from baseline at 10 mmHg) for young and older eyes as a function of IOP at ICP −5 mmHg (**C**, 3 month n = 5, 18 month n = 8), ICP 5 mmHg (**D**, 3 month n = 5, 18 month n = 6) and ICP 25 mmHg (**E**, 3 month n = 5, 18 month n = 8). (**F**) All data from panels B-D combined and plotted as a function of ONPD (mmHg).

We also compared the effect of ONPD modification on anterior retinal surface deformation. Figures 4A (3 month old) and 4B (18 month old) show group average anterior retinal surface (μm) position plotted as a function of ONPD and distance from the center of the optic nerve. In young eyes tissue deformation was apparent for locations within 300 μm of the optic nerve center. At the center of the optic nerve the highest ONPD produced on average 60 μm of backward deformation compared with baseline. For retinal locations at or near the optic nerve, anterior surface position was linearly related to the ONPD. For the lowest ONPD, which occurred with high ICP and low IOP, there was forward deformation of the anterior surface. This pattern was generally the case for the older group as shown in Fig. 4B. However, in older eyes there appeared to be less anterior surface movement with both high and low ONPD.

Data for anterior retinal surface deformation at a distance of 200 μm from the center of the optic nerve is illustrative of differences between 3 and 18 month old eyes as shown in Fig. 4C–E, for ICP levels of −5, 5 and 25 mmHg, respectively. In each case older eyes consistently showed less anterior surface deformation with IOP elevation. Statistical analysis of combined data in Fig. 4F, indicate that increased ONPD resulted in greater RNFL compression in older eyes (age effect F_1,271_ = 7.3, P = 0.008, ONPD effect F_10,271_ = 8.8, P < 0.001, interaction F_10,271_ = 1.7, P = 0.07). These combined data confirm that in 3 month old eyes anterior surface deformation showed a modest linear correlation with ONPD (r^2^ = 0.41, P < 0.001), whereas 18 month eyes showed a poorer, albeit significant correlation (r^2^ = 0.10, P < 0.001. Linear fits also confirmed that the slope for older eyes was significantly shallower than younger eyes (3 month −0.64 [95% CI, −0.77, −0.49] vs. 18 month −0.27 μm/mmHg [−0.42, −0.16], F = 12.4, P < 0.001).

### Relationship between function, blood flow and in young and older rat eyes

**Figure 5:**
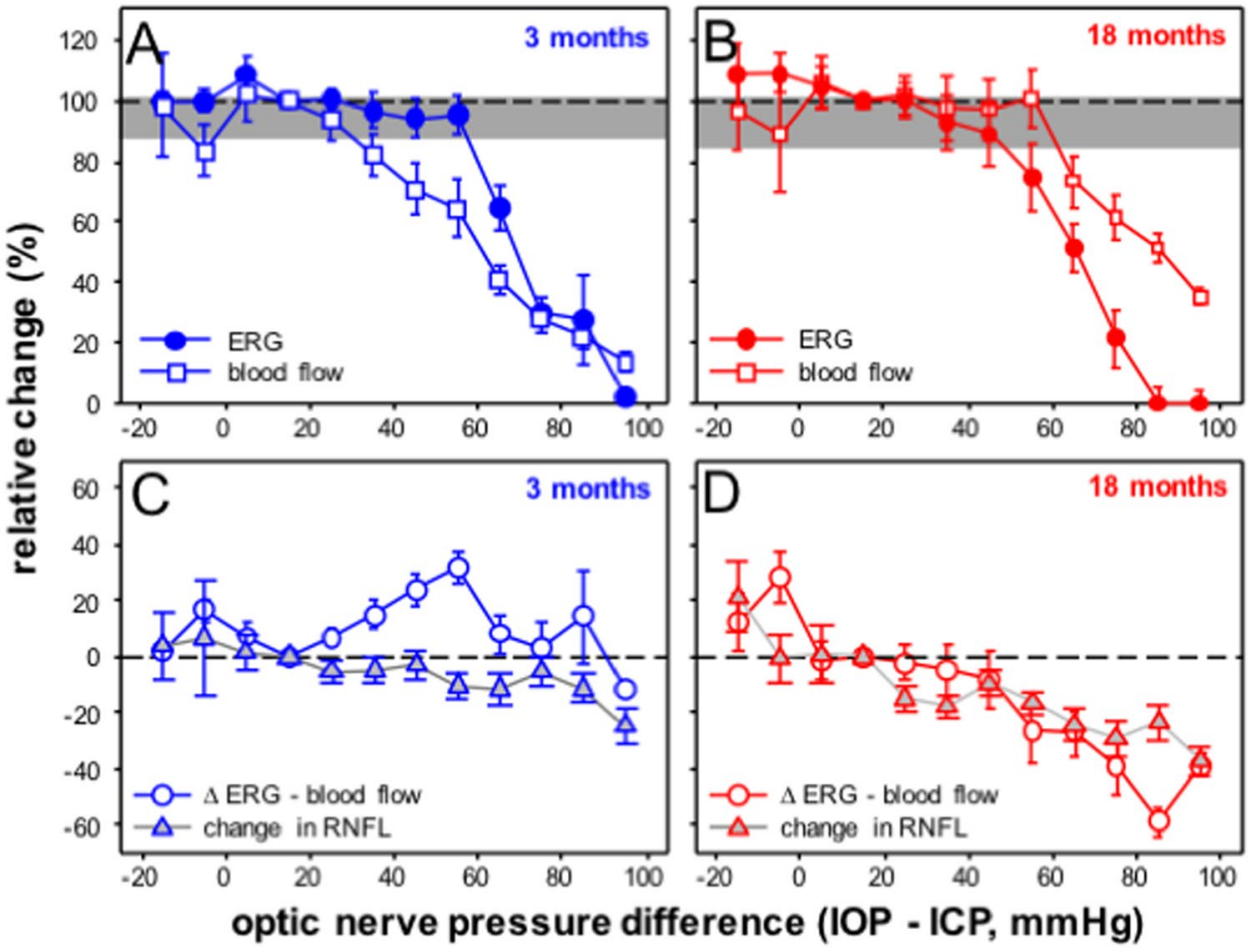
Comparisons between structure, function and blood flow across a range of optic nerve pressure differences (IOP - ICP, mmHg) in 3 and 18 month old rats. Group averaged ganglion cell function (filled circles) is overlaid on blood flow (unfilled squares) for 3 (**A**) and 18 month old groups (**B**). Grey area indicates the 95% confidence limits derived from all young and older eyes at baseline. Group averaged residuals (ERG – blood flow %, unfilled circles) are compared with % change in RNFL thickness (grey triangles) for 3 (**C**) and 18 month old groups (**D**).

How the key outcome measures changed with ONPD are compared in Fig. 5. In the 3 month old group blood flow was reduced when ONPD exceeded 35 mmHg (below baseline indicated by greyed area in Fig. 5A). In contrast, ganglion cell function was not attenuated until ONPD exceeded 65 mmHg. This would suggest that retinal function in younger eyes is buffered against mild reductions in blood flow. In comparison, ganglion cell function in older eyes declined at an ONPD of 55 mmHg. At this ONPD there was no attenuation in blood flow in older eyes (Fig. 5B). This pattern would suggest that factors other than reduced blood flow are driving the functional deficit in older rat eyes at an ONPD of 55 mmHg.

Our structural data suggests the possibility that increased RNFL axial compression may account for increased ganglion cell functional susceptibility. This is illustrated in Fig. 5C and D, where relative RNFL compression is plotted along with residuals derived by subtracting % change in blood flow from % change in ganglion cell function at each ONPD. In the 3 month old group, residuals were increased when the ONPD increased to 55 mmHg, beyond which ganglion cell function more closely followed blood flow. In contrast, residuals in older eyes decreased with higher ONPD, indicating that function declined progressively more than predicted by blood flow attenuation (Fig. 5D). Comparisons of residuals to RNFL compression suggest a closer qualitative relationship between the two parameters in older eyes (Fig. 5D) compared with younger eyes (Fig. 5C).

## Discussion

### Optic nerve pressure difference modulates both structure and function in rat eyes

We show that the rat optic nerve is both structurally and functionally sensitive to manipulation of the pressure difference across the optic nerve, by virtue of either a change in IOP or ICP. Our current data are quantitatively similar to our previous report^13^, that low ICP increased the susceptibility of retinal function to IOP elevation, whereas the converse was true of high ICP, which reduced the effect of IOP elevation (Fig. 1). Also, consistent with our previous work, we showed that retinal surface deformation (Fig. 3) and RNFL compression (Fig. 4) are linearly related to ONPD. This was in contrast to ganglion cell function, which showed a sigmoidal relationship with increasing ONPD (Fig. 1E). Thus it is likely that factors other than tissue deformation and compression fully account for ganglion cell functional integrity.

We extended our previous study, to show that blood flow through the major retinal arterioles was attenuated in a sigmoidal fashion (Fig. 2E), more akin to the relationship between ganglion cell function and ONPD. However, ONPD modification of inner retinal blood flow did not fully account for changes in ganglion cell function, as in young eyes the STR was preserved even when blood flow appeared to have been attenuated to 30% at an ONPD of 55 mmHg (Fig. 5A). We had previously posited that one explanation for this functional preservation could be an increased oxygen extraction as blood flow slows^38^. Using *in vivo* simultaneous blood flow and oxygen tension measurements we showed that when ONPD exceeded approximately 55 mmHg (i.e. IOP 60 – ICP 5 mmHg) oxygen tension was preserved despite blood flow attenuation^38^, which may be indicative of increased oxygen extraction.

### Older rat eyes are more functionally susceptible to pressure challenge

Another important observation from this study was that 18 month old rat eyes showed greater functional susceptibility to IOP elevation compared with younger 3 month old animals. This age-related deficit is consistent with other studies employing IOP elevation as a stressor in rats^39,40^ and mice^41^. In mice, Kong *et al*.^41^, using a single level of IOP elevation showed that even by 12 months there was evidence of slower functional recovery from stress, an effect that was further exacerbated at 18 months of age.

Our observation of increased functional susceptibility with age may arise from changes in connective biomechanical properties, vascular factors or age-related differences in the intrinsic capacity of ganglion cells to resist stress. Our data suggest that differences in the way that blood flow was affected by ONPD modification cannot account for the age-related increase in functional susceptibility (Fig. 2). Indeed, once we had corrected for blood pressure, older eyes appeared to show slightly better blood flow than younger eyes (Fig. 2F). This is inconsistent with the greater functional susceptibility of older eyes.

Age-related vascular changes have been strongly linked to glaucoma, as evidenced by reports of reduced vascular supply and impaired autoregulation in older eyes and in those with glaucoma^5^. Structural changes to the vasculature have been noted in 22–24 month old Wistar^42^ and Sprague-Dawley rats^43^. However, age-related differences in the vascular response to IOP elevation have not been documented. Kong *et al*.^41^ using the Heidelberg retinal flow meter showed that there was little difference between 3 and 18 month old mice in the magnitude of blood flow attenuation in response to acute IOP challenge.

Whilst our data would suggest that autoregulation was unimpaired in 18 month old rat eyes, there are several considerations. First, our Doppler OCT blood flow approach measures flow via a cross section of the large retinal arterioles and thus may fail to detect age-related deficits in smaller vessels, particularly those in intermediate and deeper layers of the retinal trilaminar network which are thought to play a larger role in autoregulation^44^. Second, the area of blood vessels assessed, whilst close to the optic nerve, may not reflect age-related differences that may exist in vessels supplying the optic nerve or in more peripheral retinal locations. However, as our functional measure encompasses all ganglion cells across the retina, it may be appropriate to make comparisons with blood flow through the major vessels by expressing both relative to baseline. Third, OCT Doppler blood flow, whilst useful for quantifying relative changes such as those induced by acute IOP elevation, is less suited for quantifying basal differences. Thus, any age-related deficit in basal blood flow would not have been highlighted. Nevertheless, having made the same relative comparisons between age groups, we are confident that poorer perfusion cannot account for our finding of age-related functional susceptibility with higher ONPD.

Another possible explanation for the disconnect between function and blood flow in older eyes is an inefficiency in oxygen extraction, as might be expected with age-related thickening of vessel walls^45^, leading to increased resistance to oxygen diffusion^46^. Impairment in the capacity of neural tissue to up regulate oxygen extraction as blood flow slows^38^, or to produce energy from oxidative phosphorylation may also help explain our observed age-related functional attenuation even though blood flow appeared to be normal. There is good evidence that older eyes have reduced mitochondrial function and increased oxidative stress^4^. Kong *et al*.^38^ have previously shown in mice that greater functional deficits and slower recovery was associated with reduced mitochondrial oxidative phosphorylation enzyme activity^41^.

### Less surface deformation but more RNFL compression in older eyes

Our data also suggest that age-related changes in the biomechanical properties of connective tissue may play a role in increasing functional susceptibility. We showed that with higher ONPD, older eyes showed less posterior deformation of the retinal surface (Fig. 4). Additionally, we observed that the relationship between RNFL thickness and ONPD was steeper in older eyes (Fig. 3). Therefore, greater RNFL compression may contribute to increased attenuation of ganglion cell function with higher ONPD in older eyes (Fig. 5D).

It has been reported that age-related changes to the peripapillary scleral and lamina cribrosa connective tissues are important contributors to ganglion cell injury in glaucoma^7^. Using a range of approaches investigators have consistently found that the lamina cribrosa^15,16^ and sclera stiffen with age in human donor eyes^17–22^. Similarly, age-related stiffening of the sclera has been observed in older non-human primate^23^ and canine eyes^24^. Age-related increase in laminar cribrosa and scleral stiffness has been attributed to changes to the extracellular matrix^18^, including altered collagen deposition, reduced elastin content and increased cross linking^47^.

A stiffer corneoscleral shell has been shown to result in larger IOP spikes^25–27^, leading to the idea that a more compliant shell can better absorb and protect the eye against IOP elevation. Our data appear to be consistent with the notion that less deformation of the retinal surface (Fig. 4) was associated with greater compression of the retinal nerve fiber layer (Fig. 3).

Why greater RNFL compression leads to acute deficits in ganglion cell function is not understood. One possibility is that greater compression leads to more activation of mechanosensors found on ganglion cells bodies and axons, for example the transient receptor potential (TRP) channels of the vanilloid subclass. Weitlauf *et al*.^48^ report that acute IOP elevation increases TRPV1 expression near ganglion cell excitatory synapses, which in turn modifies ganglion cell excitability. Whilst the role of TRPV as well as other mechanosensors in modulating ganglion cell excitability is an ongoing area of study, there is growing evidence that local mechanotransduction can modify ganglion cell excitability^49^.

We also note that while there was more RNFL compression on older eyes (Fig. 3E), there was actually less compression of the retina overall (Fig. 3F). One possibility is that this might arise from greater retinal movement toward the optic nerve in the younger more compliant optic nerve. Alternatively, the large vessels that reside in the rat optic nerve may influence our measurement of retinal thickness. With IOP elevation blood flow was slightly increased between in older eyes (Fig. 2), which may have contributed to an overestimate of total retinal thickness.

### Study limitations

Whilst the rat eye is widely used in glaucoma research, it is important to acknowledge key limitations in terms of differences in the optic nerve head anatomy. This is particularly true with regards to the lack of a well-developed collagenous lamina cribrosa, a largely glial support structure and a higher proportional area of the optic nerve head being occupied by the vasculature supplying the inner retina and choroid^50^. Although there is no laminar cribrosa per se, a pressure gradient across the optic nerve should still exist. That all our outcomes measures were influenced by changes in both IOP and ICP supports this contention. However, the relative contributions of IOP and ICP changes to ganglion cell integrity in a species lacking a collagenous lamina are likely to be different to those with a more developed lamina cribrosa. Another consequence of this anatomical difference may be that without a strong lamina the relationship between tissue deformation and ONPD is more linear, as we have observed in this study (Figs 3 and 4), rather than asymptotic as has been reported previously^12,51^. Morgan *et al*.^12^ using retinal tomography to image canine optic nerves, showed that tissue deformation reaches a maximal depth for pressure differences of approximately 15 mmHg. This non-linear relationship has also been noted in human eyes^51^. Although, rats share similarities in cerebrospinal fluid drainage pathways with humans^52,53^ their habitual supine body position differs from humans, which has implication for the optic nerve pressure difference. Thus, whilst it is useful to refine ideas in a less complex rodent model, a wider generalization of the results in terms of understanding disease pathophysiology may require studies in non-human primates.

While it would be ideal to quantify precisely the input parameters, we have employed a number of surrogate measures of the key pressures that influence the optic nerve. Specifically, we employ ventricle pressure as a surrogate for optic nerve sheath pressure and retro-laminar tissue pressure. Morgan *et al*.^54^ showed in canine that above −0.52 mmHg cerebrospinal fluid pressure and optic nerve subarachnoid space pressures is linearly related with a slope close to unity (0.99 ± 0.1). Whether this is also the case in rat has not been assessed, thus we make the assumption that changes in ICP influence optic nerve sheath pressure in a similar fashion in both young and older rats.

Our measurement of blood pressure via in indwelling cannula, remains a surrogate of true perfusion into the eye. It is known that there is a strong relationship between systemic blood pressure and retinal arterial and venous pressure in humans^55^. Kiel and Heuven^56^ showed that the effective choroidal perfusion can be described by ocular perfusion pressure (the difference between blood pressure and IOP) in rabbits. Whilst it may be reasonable to expect that pressure in rat retinal vessels is related to systemic blood pressure in a similar way, we make the assumption that this relationship is consistent in young and older rats. Figure 2F, would suggest that his assumption has validity as expressing changes in blood flow as a function of perfusion pressure difference (blood pressure - [IOP - ICP]) largely removes age-related differences in blood flow expressed against IOP (Fig. 2B–D) or against ONPD (Fig. 2E).

Although the group sizes were modest (n = 5–9), this effectively increased to n = 30–40 when we combined all ICP groups to undertake statistical comparison across age groups. Thus, we believe that our statistical outcomes are robust despite a number of sources of variability in our approach. Whilst we took care to visually confirm centration of the optic nerve position in the *en face* image immediately prior to starting the scan, there would be some variability due to the absence of image registered follow up capabilities. Variation in imaging position is likely to account for the variability in our OCT data (Fig. 3).

As already mentioned, some care should be taken in generalizing our findings regarding blood flow given the limitations of our blood flow method. Finally, whilst we would have wished to assess ganglion cell function along with structure and blood flow in the same eyes, this was not technically feasible. In order to quantify ganglion cell function, we needed absolute dark adaption, as well as placement of a light stimulator that covered the whole eye. These technical limitations precluded simultaneous OCT imaging.

## Conclusions

We find that older rat eyes showed greater functional susceptibility to elevated ONPD, regardless of whether such a difference was generated by an increase in IOP or a decrease in ICP. This functional susceptibility could not be accounted for by impaired blood flow, but was associated with greater RNFL compression in older rat eyes. These data provide insights into the mechanisms underlying age related susceptibility of retinal ganglion cells to elevated optic nerve pressure gradients. Future research in a species with a well-developed lamina cribrosa is needed to better understand the clinical significance of these findings.

## Electronic supplementary material

Supplementary material
