# Supplementary material for: Age-related changes in the response of retinal structure, function and blood flow to pressure modification in rats
Source: Sci Rep. 2018 Feb 13;8:2947. doi: 10.1038/s41598-018-21203-5 (PMC5811482; doi:10.1038/s41598-018-21203-5)
Supplement: Supplementary file 1 — Supplementary material [file 41598_2018_21203_MOESM1_ESM.pdf]

Title: Age-related changes in the response of retinal structure, function and blood flow to pressure modification in rats

Authors: Da Zhao[1], Christine TO Nguyen[1], Zheng He[1], Vickie HY Wong[1], Anna van Koeverden[1], Algis J Vingrys[1], Bang V Bui[1]

[1]Department of Optometry and Vision Sciences, University of Melbourne, Parkville, 3010, Victoria, Australia.

Correspondence: Bang V Bui

Department of Optometry and Vision Sciences, University of Melbourne, Parkville, 3010, Victoria, Australia.

Ph: +61 3 8344 7006

Email: bvb@unimelb.edu.au

SUPPLEMENTARY MATERIAL

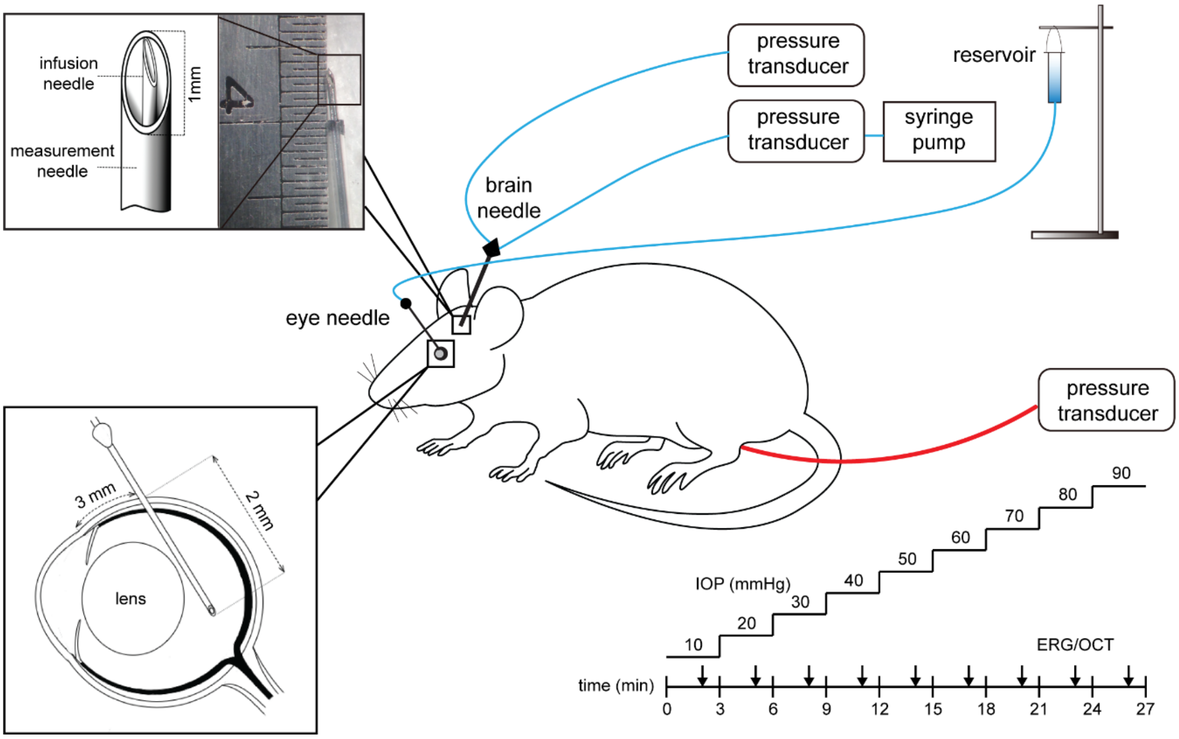

***Figure S1**. **ICP and IOP modification methodology.** ICP modification was achieved by a customized dual-lumen needle cannula place in the lateral ventricle (upper left corner). A syringe pump was used to manipulate ICP. IOP elevation was achieved via a 27G needle cannula inserted into the vitreous chamber (lower left corner) attached to a saline reservoir (upper right corner), which was placed at pre-calibrated heights. BP was continuously monitored via a femoral artery cannula. Once ICP had stabilised for 20 minutes, IOP was increased from 10 to 90 mmHg in 10 mmHg steps each lasting 3 minutes. Two minutes after the onset of each IOP step, ERG or OCT was assayed.*
